# Lipid Raft Membrane Interactivity Correlating with Cyclooxygenase-2 Selectivity of Non-Steroidal Anti-Inflammatory Drugs

**DOI:** 10.3390/membranes15090284

**Published:** 2025-09-22

**Authors:** Maki Mizogami, Hiroki Iida, Hironori Tsuchiya

**Affiliations:** 1 Anesthesiology and Pain Relief Center, Central Japan International Medical Center, Minokamo 505-8510, Gifu, Japan; h-iidamd@xqd.biglobe.ne.jp; 2 Department of Dental Basic Education, Asahi University School of Dentistry, Mizuho 501-0296, Gifu, Japan; tsuchi-hiroki16@dent.asahi-u.ac.jp

**Keywords:** cyclooxygenase-2 selectivity, lipid raft, membrane interactivity, non-steroidal anti-inflammatory drug

## Abstract

The primary mechanism of non-steroidal anti-inflammatory drugs (NSAIDs) is inhibition of prostaglandin production mediated by cyclooxygenase. Given the possible association of cyclooxygenase-2, but not cyclooxygenase-1, with membrane lipid rafts, we assessed whether the lipid raft membrane interactivity of NSAIDs correlates with cyclooxygenase-2 selectivity. Lipid raft model membranes and reference membranes were prepared with 1,2-dioleoylphosphatidylcholine/sphingomyelin/cholesterol and 1,2-dipalmitoylphosphatidylcholine, respectively. After treating the membranes with 2–50 μM NSAIDs at pH 7.4, 6.5, and 5.5, fluorescence polarization was measured to determine their membrane interactivity. Conventional NSAIDs (diclofenac, ibuprofen, indomethacin, aspirin, and flurbiprofen) and Coxibs (lumiracoxib, etoricoxib, celecoxib, valdecoxib, and rofecoxib) decreased membrane fluidity, whereas Oxicams (meloxicam, piroxicam, tenoxicam, and lornoxicam) increased. Membrane effects of NSAIDs were so dependent on medium pH that they significantly increased with reducing pH from 7.4 to 5.5. Under inflammatory acidic conditions, the lipid raft membrane interactivity of NSAIDs was more likely to correlate with cyclooxygenase-2 selectivity than the reference membrane interactivity. It is hypothesized that NSAIDs may interact with lipid raft membranes to induce membrane fluidity changes with the potency corresponding to cyclooxygenase-2 inhibition, disrupting the structural and functional integrity of lipid rafts to affect the activity of cyclooxygenase-2 localized in lipid rafts, resulting in cyclooxygenase-2 selective inhibition.

## 1. Introduction

Non-steroidal anti-inflammatory drugs (NSAIDs) are among the most widely used drugs for treatments of pain, fever, and inflammation. During the pandemic of COVID-19, their consumption significantly increased all over the world to manage symptoms of patients infected with SARS-CoV-2 [1]. Their primary mode of action has been recognized as the inhibition of prostaglandin production mediated by cyclooxygenase (COX), which has distinct isoforms, constitutively expressed COX-1 relevant to physiological functions and inducible COX-2 upregulated under pathological conditions. Although NSAIDs share the mode of action, they are different in pharmacological profiles and chemical structures.

**Figure 1:**
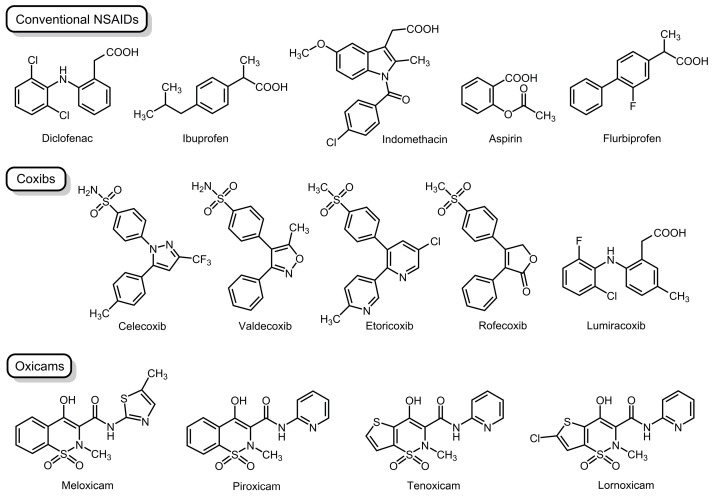
Non-steroidal anti-inflammatory drugs examined in the present study.

NSAIDs, consisting of diverse drugs, are classified according to differences in chemical structures, pharmacological/pharmacokinetic properties, and COX selectivity [2,3,4]. They are structurally classified into conventional drugs (commonly containing a carboxylic acid moiety), Coxibs (diaryl heterocyclic acid derivatives with some exceptions), and Oxicams (characterized by the enolic acid structure), as shown by representative NSAIDs in Figure 1.

In terms of COX selectivity, NSAIDs are divided into drugs selective for COX-1 or COX-2 and drugs without selectivity for either COX-1 or COX-2. While it is no doubt that COX-2 selective NSAIDs inhibit the activity of COX-2 more significantly than that of COX-1, a question arises as to how such COX-2 selectivity is produced. In addition to the direct action on COX proteins, amphiphilic NSAIDs are able to act on membrane lipids specifically surrounding the relevant protein structure [5,6,7].

Functional membrane proteins like drug-targeting enzymes are embedded in or bound to biomembrane lipid bilayers for exhibiting their intrinsic activities. Since COX-2 is the monotopic membrane protein, an understanding of its presence in plasma membranes would give a new insight into the mechanism underlying COX-2 inhibitory effects of NSAIDs. Plasma membranes are laterally heterogenous as specific lipids cluster to form nanoscopic membrane domains—lipid rafts or caveolae—which provide enzymes with a platform. Lipid rafts are cholesterol-/sphingolipid-enriched liquid-ordered domains that are distinct from surrounding liquid-disordered membrane lipid structures, whereas caveolae are a subset of lipid rafts structurally maintained or aggregated by characteristic protein caveolins [8]. Although caveolin-containing and caveolin-not-containing lipid rafts may not be functionally the same, lipid rafts and caveolae have overlapping functions [9]. It was revealed that COX-2 and caveolin are co-localized in caveolae [10,11] and COX-2 is localized in the caveolae-like structures of plasma membranes [12]. Membrane lipid rafts are also responsible for COX-2 expression in human neuroblastoma cells [13]. Several NSAIDs of therapeutic concentrations were found to influence Ras protein nanoclustering in plasma membranes and affect the organization of raft-like ordered lipid bilayers [14].

COX-2 inhibition can be firstly interpreted by a conventional mechanism in which NSAIDs directly interact with COX-2 enzyme proteins based on distinct COX affinities [15,16,17]. Given the possible association of COX-2, but not COX-1, with lipid raft domains, NSAIDs are speculated to interact with membrane lipid rafts as a drug target [18]. Therefore, we assessed whether the lipid raft membrane interactivity of structurally different NSAIDs correlated with cyclooxygenase-2 selectivity. Plasma membranes of mammalian cells are the lipid bilayers composed of diverse types of proteins and lipids. In the present study, protein-free model membranes were used to focus on the interactions between NSAIDs and membrane lipid components [19]. In order to mimic the lipid raft membranes for embedding integral membrane proteins [20], we prepared the simplified membrane lipid raft models [6], in which liquid-ordered domains were composed of cholesterol and sphingomyelin (SM) and were surrounded by a liquid-disordered non-raft membrane matrix enriched in phosphatidylcholine [21]. The tested NSAIDs were conventional drugs (diclofenac, ibuprofen, indomethacin, aspirin, and flurbiprofen), Coxibs (celecoxib, rofecoxib, valdecoxib, etoricoxib, and lumiracoxib), and Oxicams (piroxicam, meloxicam, tenoxicam, and lornoxicam), as shown in Figure 1, including drugs currently available and drugs withdrawn from the market due to their severe side effects, to compare membrane interactivity and COX-2 selectivity.

## 2. Materials and Methods

### 2.1. Chemicals

Conventional NSAIDs and Coxibs, and Oxicams were purchased from Wako Pure Chemicals (Osaka, Japan) and Tokyo Chemical Industry (Tokyo, Japan), respectively. 1,2-dipalmitoylphosphatidylcholine (DPPC), 1,2-dioleoylphosphatidylcholine (DOPC), and porcine brain SM were obtained from Avanti Polar Lipids (Alabaster, AL, USA); cholesterol from Wako Pure Chemicals; and diphenyl-1,3,5-hexatriene (DPH) from Molecular Probes (Eugene, OR, USA). 4-(2-hydroxyethyl)-1-piperazineethanesulfonic acid (HEPES) buffer (pH 7.4) and McIlvaine buffer (pH 6.5 and 5.5) were prepared to contain 125 mM NaCl and 25 mM KCl by using reagent products (Wako Pure Chemicals). Dimethyl sulfoxide (DMSO) of spectroscopic grade (Kishida; Osaka, Japan) was used for preparing reagent solutions. All other chemicals were of the highest grade available commercially.

### 2.2. Preparation of Lipid Raft Membranes and Reference Membranes

Model membranes, which were subjected to fluorescence polarization after the reactions with NSAIDs, were prepared according to our previous method [7,19]. They were labeled with a fluorescent probe DPH that has been most widely used to study the membrane interactions of NSAIDs compared with other probes [7,22,23]. The ethanol injection method [24] was used to produce unilamellar vesicles or single bilayer liposomes [25] for lipid raft membranes and biomimetic membranes. The dry film of phospholipids and cholesterol was dissolved with an ethanolic solution of DPH. An aliquot (250 μL) of the resulting solution (total lipids of 10 mM and DPH of 50 μM) was rapidly injected four times into 199 mL of HEPES buffer (pH 7.4) or McIlvaine buffer (pH 6.5 and 5.5) under stirring at 50 °C (above the phase transition temperatures of phospholipids) [26]. The membrane lipid composition was 33.3 mol% DOPC, 33.3 mol% SM, and 33.3 mol% cholesterol for lipid raft membranes [27] and 100 mol% DPPC for reference DPPC membranes.

### 2.3. Determination of Membrane Interactivity

NSAIDs dissolved in DMSO were added to the membrane preparations so that final concentrations were 2–50 μM. The concentration of DMSO was adjusted to be less than 0.5% (*v*/*v*) of the total volume so as not to affect intact membrane fluidity. Control experiments were conducted by adding an equivalent volume of DMSO. The membrane interactivity of NSAIDs was evaluated by their induced changes in membrane fluidity, which has been referred to as a determinant for the integrity of biomembranes and a modulator for the functions of membrane-embedded proteins. After reactions at 37 °C for 45 min, DPH fluorescence polarization was measured at 360 nm for excitation wavelength and 430 nm for emission wavelength by an FP-777 spectrofluorometer (Japan Spectroscopic Cooperation; Tokyo, Japan) equipped with a polarizer and a cuvette thermo-controlled at 37 °C (Shimadzu; Kyoto, Japan). Although Oxicams and indomethacin have intrinsic fluorescence properties, the used emission wavelength differs greatly from the emission maxima of Oxicams [28] and indomethacin [29], indicating that intrinsic fluorescence of NSAIDs is very unlikely to influence DPH polarization at the tested drug concentrations [30]. Polarization values were calculated by the formula (*I*_VV_ − *GI*_VH_)/(*I*_VV_ + *GI*_VH_), where *I* is the fluorescence intensity and the subscripts V and H refer to the vertical and horizontal orientation of excitation and emission polarizer, respectively. The grating correction factor (*G* = *I*_HV_/*I*_HH_) was used to correct the polarizing effects of a monochromator. Compared with controls, an increase and a decrease in fluorescence polarization indicated a decrease and an increase in membrane fluidity, respectively. When comparing the membrane interactivity between different membranes or between different conditions, the polarization changes (%) relative to control polarization values were used because the polarization values of intact membranes significantly vary depending on lipid composition and medium pH.

### 2.4. Statistical Analysis

Data were statistically analyzed by one-way ANOVA with a Bonferroni post hoc comparison using SPSS version 22 (IBM Corporation; Chicago, IL, USA). The results are expressed as means ± SD (*n* = 8 for each experiment), and values of * *p* < 0.05 and ** *p* < 0.01 were considered statistically significant.

### 2.5. Assessment of Cyclooxygenase Selectivity

To assess COX selectivity of NSAIDs, a literature search was conducted in databases PubMed and Google Scholar. Keywords NSAID, cyclooxygenase, COX-1, COX-2, and inhibition were used to retrieve relevant articles. Collected articles were reviewed by text for relevance and 50% inhibitory concentrations (IC_50_) for COX-1 and COX-2 were obtained. Mean values of log (IC_50_ for COX-1/IC_50_ for COX-2) were calculated to assess COX-2 selectivity.

## 3. Results

### 3.1. Cyclooxygenase-2 Selectivity of Non-Steroidal Anti-Inflammatory Drugs

**Figure 2:**
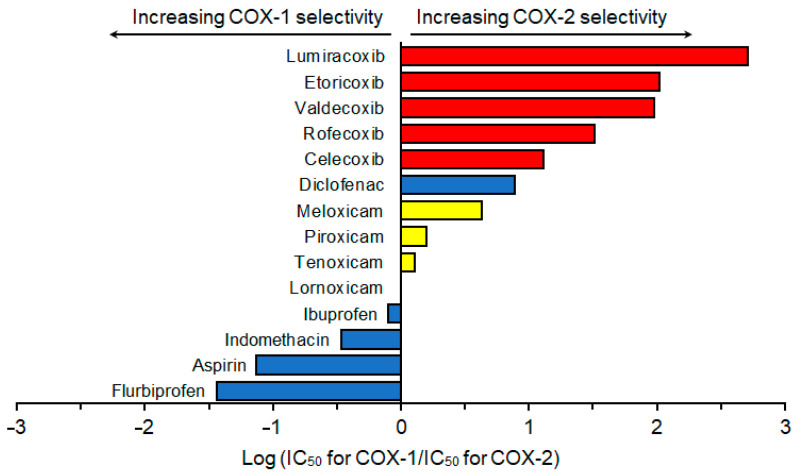
Cyclooxygenase selectivity of non-steroidal anti-inflammatory drugs, which was assessed by using the results of human whole blood assays reported previously [16,31,32,33,34,35,36,37].

Althoug a number of studies reported inhibitory effects of NSAIDs on COX-1 and COX-2, they significantly varied depending on analytical methods and samples used for the COX activity measurement and from species to species [15]. Therefore, COX selectivity was assessed by using the results of human whole blood assays [16,31,32,33,34,35,36,37]. COX selectivity of different NSAIDs is shown in Figure 2.

Based on COX selectivity, NSAIDs were classified into highly selective COX-2 inhibitor Coxibs: lumiracoxib, etoricoxib, valdecoxib, rofecoxib, and celecoxib; mildly to moderately selective COX-2 inhibitor Oxicams: meloxicam, piroxicam, tenoxicam, and lornoxicam; and less selective COX-2 (COX-1 selective) inhibitors: ibuprofen, indomethacin, aspirin, and flurbiprofen. Despite being one of the conventional NSAIDs, diclofenac had high selectivity for COX-2 that was higher than that of meloxicam but lower than that of celecoxib.

### 3.2. Effects of Non-Steroidal Anti-Inflammatory Drugs on Different Membranes

NSAIDs with different COX selectivity were subjected to the interactions with lipid raft membranes and DPPC membranes at different pH (7.4 and 5.5). They changed the fluidity of both membranes as shown in Table 1. When comparing at an identical concentration of 50 μM for each, the effect to decrease lipid raft membrane fluidity at pH 7.4 was most potent in lumiracoxib, followed by etoricoxib, celecoxib, diclofenac, valdecoxib, rofecoxib, ibuprofen, aspirin, indomethacin, and flurbiprofen. In contrast, meloxicam, piroxicam, tenoxicam, and lornoxicam increased the fluidity of lipid raft membrane in decreasing order of potency. NSAIDs induced larger fluidity changes in lipid raft membranes than in DPPC membranes. The effect of flurbiprofen to decrease lipid raft membrane fluidity was significant at pH 5.5, but not at pH 7.4. Diclofenac and celecoxib were characterized by larger increases of the lipid raft membrane interactivity at acidic pH compared with other drugs.

### 3.3. Relation Between Membrane Interactivity and Cyclooxygenase-2 Selectivity

**Figure 3:**
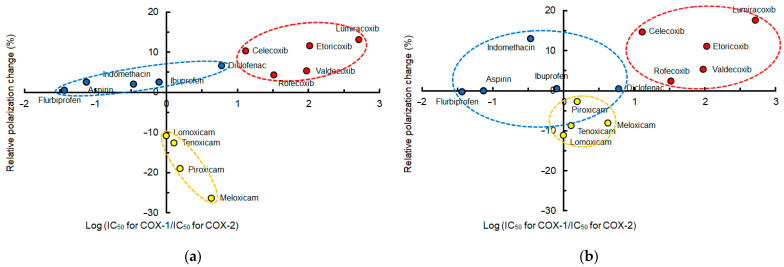
Membrane interactivity and cyclooxygenase-2 selectivity at pH 7.4 of non-steroidal anti-inflammatory drugs (50 μM for each). (**a**) Interactions with lipid raft membranes; (**b**) interactions with DPPC membranes.

In order to verify the relation between membrane interactivity and COX-2 selectivity, mean polarization changes of different membranes induced by NSAIDs at pH 7.4 were plotted against COX-2 selectivity indicated by log (IC_50_ for COX-1/IC_50_ for COX-2). Compared with the DPPC membrane interactivity, the lipid raft membrane interactivity of conventional NSAIDs (ibuprofen, indomethacin, aspirin, flurbiprofen, and diclofenac) was more likely to be localized in or converge to the same area as shown in Figure 3. Oxicams (meloxicam, piroxicam, tenoxicam, and lornoxicam) and Coxibs (lumiracoxib, etoricoxib, celecoxib, valdecoxib, and rofecoxib) showed the same convergence tendency.

**Figure 4:**
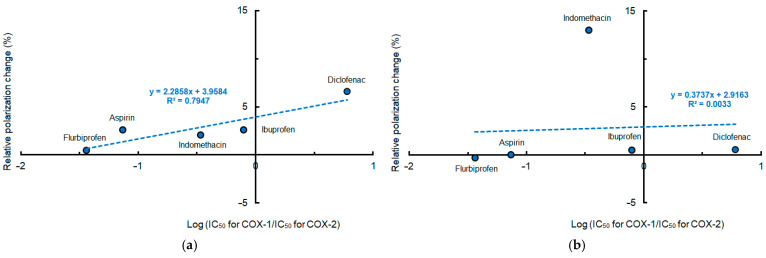
Relation between membrane interactivity at pH 7.4 and cyclooxygenase-2 selectivity of conventional non-steroidal anti-inflammatory drugs (50 μM for each). (**a**) Interactions with lipid raft membranes; (**b**) interactions with DPPC membranes.

When plotting mean polarization changes of different membranes induced by conventional NSAIDs at pH 7.4 against COX-2 selectivity indicated by log (IC_50_ for COX-1/IC_50_ for COX-2), the lipid raft membrane interactivity of diclofenac, ibuprofen, indomethacin, aspirin, and flurbiprofen was more likely to correlate with their selectivity for COX-2 than the DPPC membrane interactivity, as shown in Figure 4.

**Figure 5:**
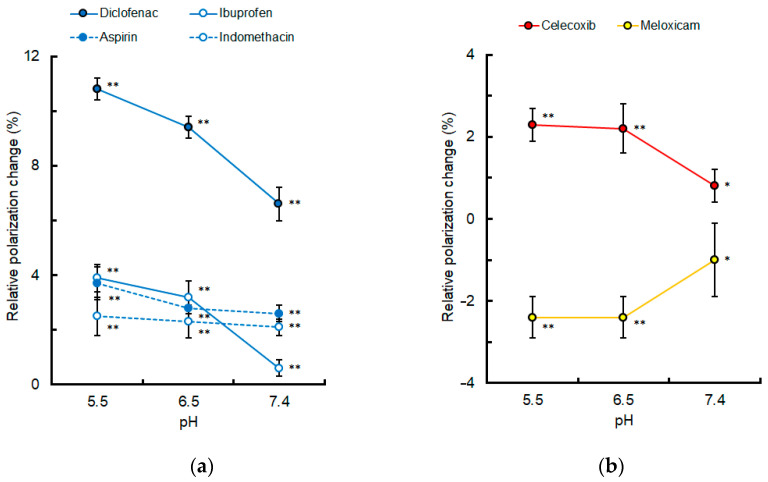
Lipid raft membrane interactivity of non-steroidal anti-inflammatory drugs at different pH. (**a**) Conventional drugs (50 μM for each); (**b**) celecoxib and meloxicam (2 μM for each). Values are mean ± SD (*n* = 8). * *p* < 0.05 and ** *p* < 0.01 compared with controls.

Since pH is reduced to 5.5–6.5 in inflamed tissues [38,39], the lipid raft membrane interactivity of selected NSAIDs was compared at pH 7.4, 6.5, and 5.5, as shown in Figure 5. The effects of diclofenac, ibuprofen, aspirin, and indomethacin (50 μM for each) to decrease membrane fluidity were increased with reducing pH. The effects of celecoxib (2 μM) to decrease membrane fluidity and the effects of meloxicam (2 μM) to increase membrane fluidity were also increased with reducing pH.

**Figure 6:**
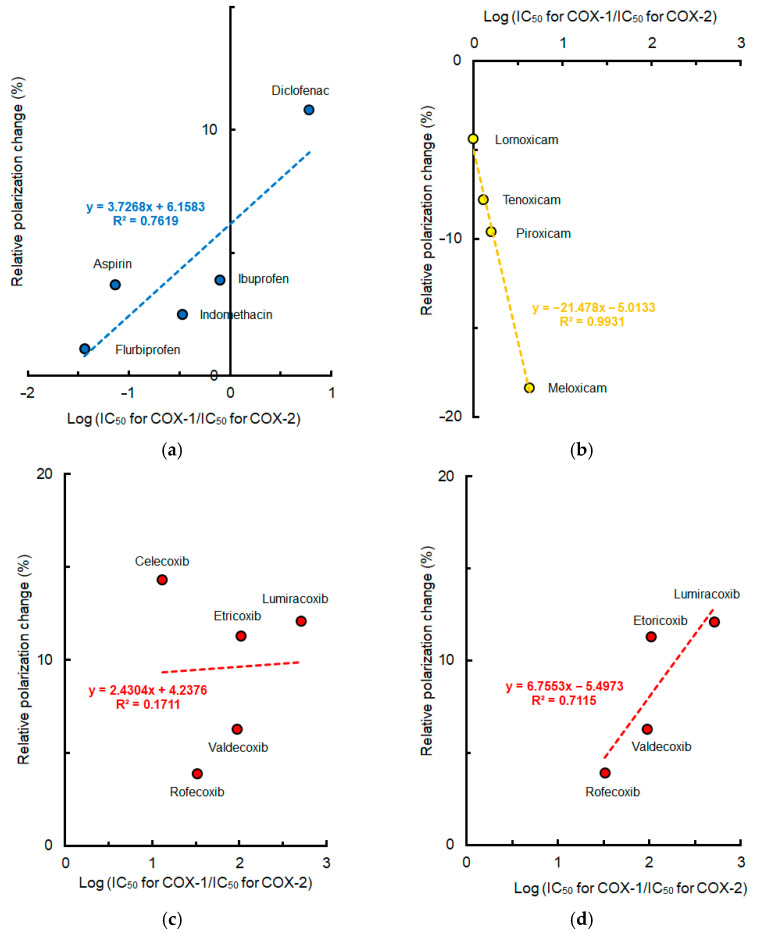
Relation between lipid raft membrane interactivity at pH 5.5 and cyclooxygenase-2 selectivity of non-steroidal anti-inflammatory drugs (50 μM for each). (**a**) Conventional drugs; (**b**) Oxicams; (**c**) Coxibs; (**d**) Coxibs other than celecoxib.

Mean polarization changes of lipid raft membranes induced by NSAIDs at pH 5.5 were plotted against COX-2 selectivity indicated by log (IC_50_ for COX-1/IC_50_ for COX-2). The lipid raft membrane interactivity of conventional NSAIDs and Oxicams seemed to correlate with their COX-2 selectivity as shown in Figure 6. As to Coxibs, celecoxib exceptionally produced greater membrane effects at acidic pH compared with other drugs. By excluding celecoxib, however, the lipid raft membrane interactivity of Coxibs became increasingly likely to correlate with their COX-2 selectivity.

## 4. Discussion

In order to propose the hypothetical COX-2 selective mechanism of NSAIDs, we comparatively assessed the membrane interactions of structurally different NSAIDs with distinctly different COX-2 selectivity at pH 7.4, 6.5, and 5.5 by using protein-free model membranes. Although there is a limitation in their direct extrapolation to actual cells, our main findings are as follows: (1) while the tested drugs interact with membranes to change their fluidity at low micromolar concentrations, their effects are greater on lipid raft membranes than DPPC membranes with only a few exceptions; (2) based on the membrane interactivity, the tested drugs are classified into conventional NSAIDs to decrease membrane fluidity mildly to moderately (with the exception of diclofenac to decrease significantly), Coxibs to decrease membrane fluidity significantly, and Oxicams to increase membrane fluidity significantly; (3) the drug and membrane interactions are so pH-dependent that reducing the pH increases the lipid raft membrane interactivity of NSAIDs, particularly diclofenac and celecoxib; and (4) the lipid raft membrane interactivity of NSAIDs at pH 5.5 could correlate with their COX-2 selectivity when excluding celecoxib.

Although both COX isozymes are monotopically inserted into membrane lipid bilayers [40], COX-1 and COX-2 integrate distinctively with the different lipid membranes [41]. Given the localization of COX-2 in lipid rafts or caveolin-containing lipid rafts [10,11,12] and the effects of NSAIDs on raft-like membrane compartments [14], it is speculated that membrane-interacting NSAID structures specifically disrupt the integrity of lipid rafts to affect the activity of COX-2, thereby producing COX-2 selectivity depending on drug structures. Membrane lipid rafts with the pathogenetic contribution can be a therapeutic target for drugs [42].

In the present study, NSAIDs were subjected to the interactions with lipid raft membranes and DPPC membranes at physiological and acidic pH. Consequently, conventional NSAIDs and Coxibs were revealed to decrease membrane fluidity, but Oxicams to increase membrane fluidity. These results agree with previous studies that investigated the interactions of NSAIDs with liposomal and cellular membranes and demonstrated the membrane fluidity-decreasing effects of conventional NSAIDs and celecoxib [7,43,44,45,46] and the membrane fluidity- and permeability-increasing effects of Oxicams [47,48]. In other studies [49,50,51,52], however, diclofenac, ibuprofen, aspirin, and celecoxib increased the fluidity of membranes prepared with phosphatidylcholine or phosphatidylcholine plus cholesterol at pH 7.4. Such a discrepancy in the membrane effects of conventional NSAIDs and Coxibs may be attributable to different concentrations of the tested drugs that our study assessed the membrane interactivity at low micromolar concentrations, but the other studies at much higher concentrations. The membrane interactivity of NSAIDs is so dependent on their concentrations that NSAIDs that decrease the membrane fluidity at relatively low concentrations conversely increase the membrane fluidity with increasing concentrations to high micromolar levels [7].

Pharmacological effect and distribution efficiency in the body of membrane-acting drugs are evaluated by their partition coefficient and hydrophobicity or lipophilicity. Partition coefficient is expressed by log P or log C_o_/C_a_, where C_o_ and C_a_ are concentrations of drugs in the organic phase (organic solvent) and in the aqueous phase (water or buffer) at the state of equilibrium. Since log P (octanol/water) is 0.7 for meloxicam, −0.14 for piroxicam, and −0.75 for tenoxicam [53], they partition in 1,2-dimyristoylphosphatidylcholine membrane bilayers more efficiently in this order [54]. Oxicams with higher partition capacity in a liposome/water system (piroxicam > tenoxicam > meloxicam > lornoxicam) conversely show smaller ability to increase the membrane fluidity of egg yolk phosphatidylcholine liposomes at pH 7.4 (lornoxicam > meloxicam > tenoxicam > piroxicam) [47]. The decreasing order of log P (octanol/water) is 4.06–4.36 for diclofenac, >3.72–4.10 for ibuprofen, >2.60–3.11 for indomethacin, and 1.19–1.39 for aspirin [55]. Celecoxib and rofecoxib have log P (octanol/water) of 3.68 and 1.71, respectively [56]. Lipophilicity is evaluated to be celecoxib > etoricoxib > valdecoxib > rofecoxib > piroxicam > meloxicam > tenoxicam [57]. These relative properties do not necessarily agree with the present results on the lipid raft membrane interactivity and the DPPC membrane interactivity of NSAIDs.

The membrane effects of drugs are determined by their partitioning and deeper location in membrane lipid bilayers. Incorporation of drugs to the deep regions of membranes depends on the form of drug molecules and the membrane surface charge. Acid dissociation constant (p*K*_a_) significantly influences the membrane interaction of NSAIDs. Inflammatory microenvironments are characterized by acidosis with extracellular pH values ranging from 5.5 to 7.0, as reported for synovial fluids from the joints of patients with rheumatoid arthritis, inflamed tissues caused by bacterial infection, and atherosclerotic plaque [38,39]. Neutrophil metabolism shifts toward aerobic glycolysis in inflamed tissues [39] and lactate production is elevated in the complete Freund’s adjuvant-induced arthritis [58], resulting in inflammatory acidification. In the present study, we assessed the membrane interactivity of NSAIDs at pH 5.5 and 6.5 to simulate inflammatory conditions, in addition to physiological pH 7.4. The tested conventional NSAIDs with a carboxylic acid moiety have p*K*_a_ values ranging from 3.5 of aspirin to 4.9 of ibuprofen [59] and Oxicams comprising enolic acid derivatives have p*K*_a_ values between 4.2 and 5.3 [60], whereas Coxibs being diaryl heterocyclic compounds have p*K*_a_ of varying values. Since most NSAIDs are weak acids with p*K*_a_ values of 3.5–5.5, their molecules are exclusively present in an ionized (anionic) form at pH being >p*K*_a_ and preferentially remain on the membrane surface regions under physiological conditions by electrostatically interacting with phospholipid headgroups. Reducing pH leads to an increase in the ratio of non-ionized (neutral) to ionized molecules, increasing drug lipid solubility [53]. When pH is <p*K*_a_, NSAIDs are mostly present in non-ionized form. Therefore, they can efficiently penetrate the deeper membrane regions and hydrophobically interact with phospholipid acyl chains to induce more significant changes in membrane fluidity. The effects of pH reduction on the NSAID and membrane interactions were different between lipid raft membranes and DPPC membranes, which is attributed to a difference in membrane lipid composition that significantly affects the membrane interactivity of drugs [7,54]. Under inflammatory acidic conditions of pH 5.5, the lipid raft membrane interactivity of NSAIDs correlates with their COX-2 selectivity at relatively low concentrations. Besides pH-decreasing changes, inflammation is associated with membrane lipid rafts. Lipid rafts responsible for cellular inflammatory responses are enlarged to harbor activated receptors and adaptor molecules and are abundantly present in inflammatory cells to have increased levels of enzymes, receptors, and ion channels, contributing to neuroinflammation and pain processing [61].

Celecoxib was characterized by its induced greater increases in the lipid raft membrane interactivity with reducing pH. Since celecoxib exceptionally has a large p*K*_a_ value of 11.1 [62], almost all of its molecules are in the non-ionized form even at acidic pH of 5.5 and 6.5, facilitating the interaction with lipid raft membranes. Celecoxib molecules are more readily distributed in the deeper regions of membrane lipid bilayers at pH 5.0, while remain closer to the membrane surface at pH 7.4 [52]. A fluorescent probe DPH used in the polarization analysis is located within the deeper regions of lipid bilayers, therefore the membrane effects of celecoxib may have been reflected more largely in DPH polarization changes at acidic pH.

Diclofenac is exceptionally highly selective for COX-2 in conventional NSAIDs and lumiracoxib has the highest COX-2 selectivity of Coxibs. Unlike Coxibs consisting of three 5- or 6-membered rings, lumiracoxib lacks the tricyclic structure and has neither a sulfonamide nor a sulfone group. In diclofenac, a 2′-chloro-6′-fluoroaniline moiety of lumiracoxib is replaced by 2′,6′-dichloroaniline and a methyl group is not present at the 5-position (Figure 1). The structural similarity of diclofenac and lumiracoxib may be associated with increasing both COX-2 selectivity and lipid raft membrane interactivity.

COX-2 selectivity of NSAIDs is influenced by their ability to fit into the active site of COX-2 and their binding to specific residues associated with COX-2 activity. A quantum crystallographic study on the complex formation of NSAIDs and COX enzymes indicated a significant difference in binding profiles between conventional NSAIDs and others (Coxibs and Oxicams) [63]. Besides the direct interaction with enzyme proteins, Barbato et al. [64] suggested that membrane affinity is a determinant for the specific binding of NSAIDs to COX-2. Lúcio et al. [47] related the effectiveness of Oxicams in increasing membrane fluidity to a difference in their COX-2 selectivity. These studies used phospholipid or egg yolk phosphatidylcholine liposomal membranes to characterize the membrane interactivity of NSAIDs. In contrast, we focused on lipid rafts structurally and functionally associated with monotopic membrane COX-2 and used lipid raft model membranes to compare the membrane interactivity of NSAIDs. Consequently, the interactions of NSAIDs with lipid raft membranes prepared with DOPC, SM, and cholesterol are more likely to correlate with COX-2 selectivity compared with DPPC membranes consisting of phospholipid alone. It is speculated that cholesterol and SM specific to lipid rafts are responsible for such membrane interactivity of COX-2 selective NSAIDs.

By interacting with lipid raft membranes, conventional NSAIDs and Coxibs decrease membrane fluidity, whereas Oxicams increase it. A question arises as to how NSAIDs with opposite membrane effects inhibit COX-2. The structural and functional integrity of lipid rafts is crucial for the activity of monotopic membrane proteins that utilize membrane lipid raft domains as a platform. Lipid raft disruptors to deplete SM and cholesterol decrease and increase cell membrane fluidity, respectively [65]. NSAIDs to decrease membrane fluidity would induce the liquid-ordered phase as well as SM depletors and interfere with the structural integrity of lipid rafts by disrupting the clustering of the ordered domains [66]. While green tea catechins exhibit anti-inflammatory activity, they interact with liposome and cell membranes to decrease membrane fluidity [67]. Such membrane-interacting catechins, particularly (−)-epigallocatechin-3-gallate and its derivatives, decrease the membrane fluidity of lipid raft-like liposomes to disrupt the integrity of lipid rafts [68]. Although cholesterol regulates the functioning of membrane domains and the structural integrity of lipid rafts, its depletion increases membrane fluidity to disrupt lipid rafts [69]. NSAIDs to increase membrane fluidity would induce the liquid-disordered phase as well as cholesterol depletors and affect the intrinsic ordered property of lipid rafts. While turmeric curcumin has an anti-inflammatory effect, it interacts with lipid bilayers to increase cell membrane fluidity [67]. Curcumin and its derivatives not only inhibit COX-2 expression [70] but also modulate the integrity of membrane lipid rafts [71]. It is hypothesized that disruption of the structural and functional lipid raft integrity, which is induced by membrane fluidity-decreasing and -increasing NSAIDs, could affect the activity of COX-2 localized in lipid rafts, resulting in COX-2 selective inhibition.

In addition to COX-2 inhibition through disrupting the lipid raft integrity, membrane fluidity-decreasing NSAIDs may interfere with COX-2 activation by modifying the property of membrane microenvironments surrounding the enzyme proteins. Since 5-lipoxygenase binds to nuclear membranes for activation, this monotopic membrane enzyme exclusively interacts with fluid (fluidity-increased) membranes to be activated, but not with rigid (fluidity decreased) membranes [72]. If monotopic COX-2 is similarly activated by binding to plasma membranes with the relatively high fluidity, conventional NSAIDs (particularly diclofenac) and Coxibs could inhibit the activity of COX-2 by decreasing membrane fluidity. Membrane fluidity increases by Oxicams induce the liquid-disordered phase, which could also prevent the formation of liquid-ordered domains for COX-2 to localize in and exhibit the intrinsic activity.

These are just hypothetical mechanisms speculated from the interactions of NSAIDs with lipid raft model membranes that induce membrane fluidity changes to characterize each individual drug. They essentially need to be further verified and experimentally confirmed by cellular systems. In that case, cholesterol depletors like methyl-β-cyclodextrin may be useful because they are presumed to disrupt lipid rafts and modify membrane fluidity.

## 5. Conclusions

COX-2 highly selective Coxibs and diclofenac, COX-2 mildly to moderately selective Oxicams, and COX-1 selective conventional NSAIDs have the common property to interact with lipid membranes in a membrane lipid composition-dependent manner. Under inflammatory acidic conditions, the lipid raft membrane interactivity of NSAIDs to decrease or increase membrane fluidity is more likely to correlate with COX-2 selectivity than the DPPC membrane interactivity. The localization and the expression of COX-2 are closely associated with membrane lipid rafts, but not those of COX-1. It is hypothesized that membrane-interacting NSAIDs may structure specifically disrupt the structural and functional integrity of membrane lipid rafts and affect the activity of COX-2 localized or expressed in lipid rafts, resulting in COX-2 selective inhibition. Of course, the present experiments using model membranes have a limitation as to what extent the results can be extrapolated to the COX-2 inhibitory effects of NSAIDs on actual cells. It is critical to take account of the fact that there are some differences between lipid raft membranes prepared with DOPC, SM, and cholesterol and lipid raft domains present in cell membranes. Methodologically, protein-free model membranes were used to focus on the interactions of NSAIDs with lipid components, eliminating the possible contribution of functional proteins. However, our hypothetical mechanism may be supported by a previous study that NSAIDs affecting the physicochemical property of phosphatidylcholine/cholesterol membranes can alter cholesterol-dependent nanoclustering in cell plasma membranes [73].

## Figures and Tables

**Table 1:** Effects of non-steroidal anti-inflammatory drugs on different membranes.

	Polarization Change				
Concentration (μM)	Interaction with Lipid Raft Membrane at pH 7.4				
	Diclofenac	Ibuprofen	Indomethacin	Aspirin	Flurbiprofen
50	0.0156 ± 0.0014 **	0.0062 ± 0.0007 **	0.0046 ± 0.0007 **	0.0058 ± 0.0007 **	0.0012 ± 0.0012
10	0.0047 ± 0.0009 **	0.0009 ± 0.0009	0.0019 ± 0.0011 *	0.0004 ± 0.0013	0.0004 ± 0.0016
2	0.0005 ± 0.0013	0.0003 ± 0.0014	0.0000 ± 0.0012	0.0000 ± 0.0007	0.0002 ± 0.0012
	**Lumiracoxib**	**Etoricoxib**	**Celecoxib**	**Valdecoxib**	**Rofecoxib**
50	0.0304 ± 0.0008 **	0.0257 ± 0.0005 **	0.0243 ± 0.0014 **	0.0122 ± 0.0010 **	0.0096 ± 0.0011 **
10	0.0193 ± 0.0012 **	0.0130 ± 0.0010 **	0.0127 ± 0.0007 **	0.0043 ± 0.0009 **	0.0026 ± 0.0005 **
2	0.0145 ± 0.0009 **	0.0035 ± 0.0010 **	0.0019 ± 0.0009 *	0.0005 ± 0.0009	0.0000 ± 0.0013
	**Meloxicam**	**Piroxicam**	**Tenoxicam**	**Lornoxicam**	
50	–0.0584 ± 0.0007 **	–0.0421 ± 0.0012 **	–0.0282 ± 0.0014 **	–0.0241 ± 0.0006 **	
10	–0.0147 ± 0.0009 **	–0.0040 ± 0.0007 **	–0.0058 ± 0.0009 **	–0.0055 ± 0.0008 **	
2	–0.0024 ± 0.0019 *	–0.0004 ± 0.0012	–0.0004 ± 0.0010	–0.0006 ± 0.0011	
	**Interaction with Lipid Raft Membrane at pH 5.5**				
	**Diclofenac**	**Ibuprofen**	**Indomethacin**	**Aspirin**	**Flurbiprofen**
50	0.0238 ± 0.0009 **	0.0086 ± 0.0010 **	0.0055 ± 0.0015 **	0.0083 ± 0.0013 **	0.0025 ± 0.0008 **
	**Lumiracoxib**	**Etoricoxib**	**Celecoxib**	**Valdecoxib**	**Rofecoxib**
50	0.0270 ± 0.0014 **	0.0259 ± 0.0005 **	0.0316 ± 0.0012 **	0.0140 ± 0.0007 **	0.0087 ± 0.0008 **
	**Meloxicam**	**Piroxicam**	**Tenoxicam**	**Lornoxicam**	
50	–0.0411 ± 0.0011 **	–0.0213 ± 0.0019 **	–0.0174 ± 0.0008 **	–0.0098 ± 0.0012 **	
	**Interaction with DPPC Membrane at pH 7.4**				
	**Diclofenac**	**Ibuprofen**	**Indomethacin**	**Aspirin**	**Flurbiprofen**
50	0.0010 ± 0.0002 *	0.0010 ± 0.0005 *	0.0252 ± 0.0020 **	0.0000 ± 0.0004	–0.0006 ± 0.0007
	**Lumiracoxib**	**Etoricoxib**	**Celecoxib**	**Valdecoxib**	**Rofecoxib**
50	0.0304 ± 0.0006 **	0.0194 ± 0.0012 **	0.0283 ± 0.0009 **	0.0092 ± 0.0011 **	0.0044 ± 0.0007 **
	**Meloxicam**	**Piroxicam**	**Tenoxicam**	**Lornoxicam**	
50	–0.0144 ± 0.0027 **	–0.0050 ± 0.0025 **	–0.0153 ± 0.0011 **	–0.0197 ± 0.0015 **	

Values are mean ± SD (*n* = 8). * *p* < 0.05 and ** *p* < 0.01 compared with controls.

## Data Availability

The original contributions presented in this study are included in the article. Further inquiries can be directed to the corresponding author.

## Abbreviations

The following abbreviations are used in this manuscript:

COX	Cyclooxygenase
DMSO	Dimethyl sulfoxide
DOPC	1,2-dioleoylphosphatidylcholine
DPH	Diphenyl-1,3,5-hexatriene
DPPC	1,2-dipalmitoylphosphatidylcholine
HEPES	4-(2-hydroxyethyl)-1-piperazineethanesulfonic acid
IC_50_	50% inhibitory concentration
NSAID	Non-steroidal anti-inflammatory drug
p*K*_a_	Acid dissociation constant
SM	Sphingomyelin
